# Rare single gene disorders: estimating baseline prevalence and outcomes worldwide

**DOI:** 10.1007/s12687-018-0376-2

**Published:** 2018-08-14

**Authors:** Hannah Blencowe, Sowmiya Moorthie, Mary Petrou, Hanan Hamamy, Sue Povey, Alan Bittles, Stephen Gibbons, Matthew Darlison, Bernadette Modell, Congenital Disorders Expert Group, A. H. Bittles, H. Blencowe, A. Christianson, S. Cousens, M. Darlison, S. Gibbons, H. Hamamy, B. Khoshnood, C. P. Howson, J. E. Lawn, P. Mastroiacovo, B. Modell, S. Moorthie, J. K. Morris, P. A. Mossey, A. J. Neville, M. Petrou, S. Povey, J. Rankin, L. Schuler-Faccini, C. Wren, K. A. Yunis

**Affiliations:** 1 Centre for Maternal, Adolescent, Reproductive, and Child Health, London School of Hygiene and Tropical Medicine, London, UK; 2 PHG Foundation, 2 Worts Causeway, Cambridge, UK; 3 Institute of Women’s Health, University College London, London, UK; 4 Department of Genetic Medicine and Development, Geneva University, Geneva, Switzerland; 5 University College London, London, UK; 6 School of Medical and Health Sciences, Edith Cowan University, Perth, Australia; 7 Centre for Comparative Genomics, Murdoch University, Perth, Australia; 8 Department of Geography and Environment, London School of Economics, London, UK; 9 WHO Collaborating Centre for Community Genetics, Centre for Health Informatics and Multi-professional Education (CHIME), University College London, London, UK

**Keywords:** Rare genetic disorders, Birth prevalence, Mortality, Disability

## Abstract

As child mortality rates overall are decreasing, non-communicable conditions, such as genetic disorders, constitute an increasing proportion of child mortality, morbidity and disability. To date, policy and public health programmes have focused on common genetic disorders. Rare single gene disorders are an important source of morbidity and premature mortality for affected families. When considered collectively, they account for an important public health burden, which is frequently under-recognised. To document the collective frequency and health burden of rare single gene disorders, it is necessary to aggregate them into large manageable groupings and take account of their family implications, effective interventions and service needs. Here, we present an approach to estimate the burden of these conditions up to 5 years of age in settings without empirical data. This approaches uses population-level demographic data, combined with assumptions based on empirical data from settings with data available, to provide population-level estimates which programmes and policy-makers when planning services can use.

**Electronic supplementary material:**

The online version of this article (10.1007/s12687-018-0376-2) contains supplementary material, which is available to authorized users.

## Introduction

Overall child mortality rates have shown large decreases over the past decades, in particular from reductions in deaths from infections, diarrhoea and vaccine-preventable diseases. Consequently, child mortality levels are now very low in many settings and policy attention is shifting to focus on non-communicable conditions, which now make up a larger relative proportion of all under-five deaths (Liu et al. 2016). In addition, in the Sustainable Development Goal era, strategies are increasingly seeking to move beyond survival to consider morbidity and disability outcomes, as highlighted in the Global Strategy for Women’s, Children’s and Adolescent’s Health (2016–2030) themes—Survive, Thrive, Transform (Every Woman Every Child 2017). In settings with very low levels of communicable disease mortality, genetically determined disorders make up an important proportion of both stillbirths and child mortality, and ongoing disability. Genetically determined disorders can be divided into two broad groups: ‘single gene disorders’ caused by gene variants with strong effect and ‘genetic risk factors’—gene variants with weaker effect causing disease only when combined with other genetic and/or environmental factors*.*

Single gene disorders arise in the first place from gene mutation. Since this can occur in any gene, single gene disorders can affect any aspect of structure or function and they are extraordinarily diverse (McKusick-Nathans Institute of Genetic Medicine 2017). Despite their clinical diversity, single gene disorders have a common biological basis, all have the potential to be passed on to offspring and all require the same basic genetic and management services. These include accurate diagnosis, risk assessment and information for the affected individual and their family, and access to options for managing risk and services for affected children.

The Modell Global Database of Congenital Disorders MGDb uses a set of defined methods to relate demographic data to the known birth prevalence of selected groups of congenital disorders, in order to generate estimates relevant to public health, policy-making and clinical practice (Modell et al. 2017). For the purpose of MGDb, single gene disorders are divided into two groups: firstly, ‘rare single gene disorders’, where the birth prevalence can be predicted from the balance between the rate at which disease gene variants arise by new mutation, and the rate at which they are lost because affected individuals die or fail to reproduce (Haldane 1949; Harris 1970; Cavalli-Sforza and Bodmer 2013); secondly, ‘common single gene disorders’ when the frequency in the population is increased as the causative gene variant confers a selective advantage in the local environment (e.g. the sickle cell gene providing protection against malaria) and country-specific information is necessary to define their birth prevalence. In addition, selected ‘genetic risk factors’ that can have an impact on early life mortality and morbidity are included in MGDb (Bhutani et al. 2013; Smits-Wintjens et al. 2008).

This paper is the sixth in this special issue on methods for estimating the global burden of congenital disorders. Here, we describe the methods used in the MGDb to estimate the collective baseline birth prevalence of rare single gene disorders, and the effect of available interventions on affected birth prevalence and outcomes. Methods to estimate ‘genetic risk factors’ or ‘common single gene disorders’ are not discussed in this paper. Further details of genetic risk factors included in MGDb (rhesus negativity, G6PD deficiency and alpha plus thalassaemia) and common single gene disorders (e.g. haemoglobin disorders, cystic fibrosis, oculo-cutaneous albinism) can be accessed online (Modell et al. 2017).

## Methods

The inheritance of rare single gene disorders generally follows Mendelian inheritance patterns. They include autosomal dominant conditions, autosomal recessive and X-linked disorders. In MGDb since the overall aim is to support policy-making in maternal and child health, only early-onset rare single gene disorders are considered. For the purposes of MGDb, these disorders are grouped as ‘early-onset dominant disorders’, ‘recessive disorders’, ‘X-linked disorders’ and ‘genetic type unknown’ (Table 1). Later-onset single gene disorders such as family cancer syndromes, adult polycystic disease of the kidney or familial hypercholesterolaemia are not included.

**Table 1:** Overview of rare single gene disorders include in Modell Global Database

Inheritance	Terminology of groupings of disorders used in MGDb
Autosomal dominant	*Early-onset dominant disorders* manifest at or soon after birth, are often due to new mutation, and usually lead to early death or seriously impaired reproductive fitness. Most occur without a family history, and are likely to be eliminated by natural selection in the first generation in the absence of diagnosis and care. Examples include osteogenesis imperfecta, achondroplasia and Apert’s syndrome.
Autosomal Recessive	*Recessive disorders* occur in one in four of the offspring of couples who both carry a potentially lethal variant of the same gene. Most people are asymptomatic carriers of at least one potentially lethal gene variant, but as most gene variants are individually rare in the general population, the chance of an at-risk union is low. Therefore, most recessive disorders are uncommon, and most affected infants are born to parents without an antecedent family history. Examples include cystic fibrosis and glycogen storage disorders. Parental consanguinity increases the chances that a couple will both carry the same recessive gene variant and hence is associated with an increased birth prevalence of recessive single gene disorders. In MGDb the term ‘consanguinity-associated disorders’ is used to refer to this increment, and they are grouped separately because their birth prevalence depends on the population prevalence of parental consanguinity (Bittles and Black 2010b; Sheridan et al. 2013).
X-linked	*X-linked disorders* are typically transmitted by unaffected female carriers and expressed in 50% of their male offspring. Female carriers may be affected to varying degrees; female homozygotes are rare. They tend to present during childhood and there is often a family history. Examples include haemophilia, Duchenne muscular dystrophy and X-linked mental retardation.
Genetic type unknown	*Genetic type unknown disorders* for which the type of inheritance (e.g. dominant, X-linked, recessive) is not known.

**Fig. 1:**
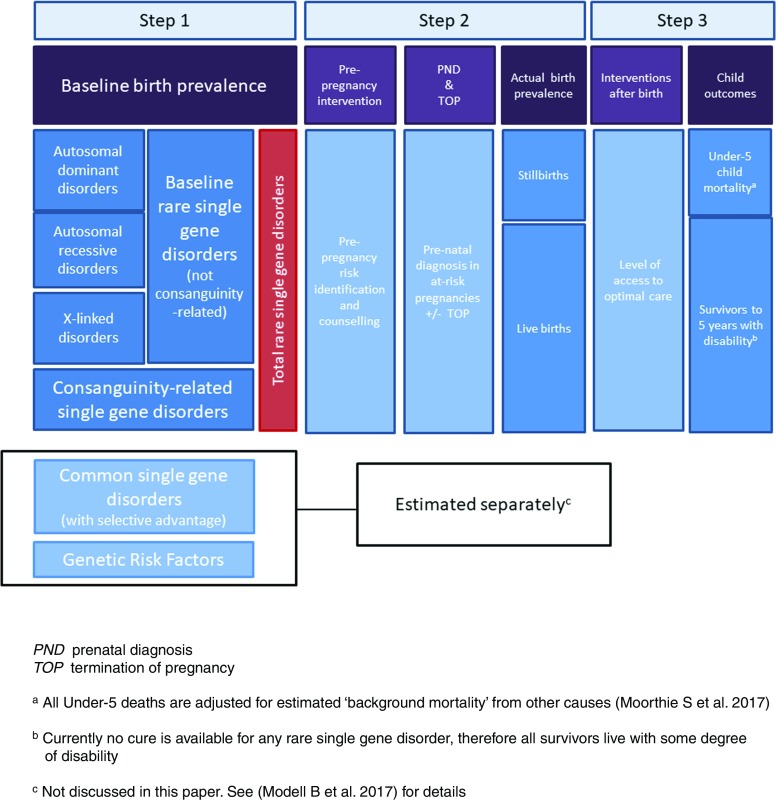
Overview of estimation of rare single gene disorders in MGDb

As with other conditions modelled in MGDb, the first step is to estimate the baseline birth prevalence of single gene disorders, in the absence of any interventions (Fig. 1). Baseline or potential birth prevalence includes stillbirths and livebirths, but excludes miscarriages. MGDb follows the European Congenital Anomalies Registry (EUROCAT) convention and uses ‘fetal death’ (death in utero after 20 weeks’ gestation) as a proxy for stillbirth and all losses before 20 weeks’ gestation are viewed as miscarriages (European Surveillance of Congenital Anomalies (EUROCAT)). In keeping with ICD-10, regardless of gestation, all births with any signs of life following separation from the mother are counted as livebirths (World Health Organization 2010).

The global prevalence of rare single gene disorders is estimated in MGDb using data on the birth prevalence of these disorders from populations with available data and applying these estimates to populations currently lacking such data, with adjustments where necessary.

### Step 1—Estimation of baseline birth prevalence

Since birth prevalence of rare single gene disorders with no selective advantage reflects a balance between new mutation and loss due to natural selection, we considered factors that could affect this balance. These include the effect of advanced paternal age on mutation rate and of parental consanguinity on affected birth prevalence and thus on selection pressure.

We estimated baseline birth prevalence separately for non-consanguinity- and consanguinity-associated disorders. The overall baseline birth prevalence of rare single gene disorders is estimated as the baseline prevalence of non-consanguinity-associated disorders plus the baseline prevalence of consanguinity-associated disorders.

#### 1a—Estimation of non-consanguinity-related baseline rare single gene disorders prevalence

##### Observational data of birth prevalence of early-onset single gene disorders and adjustments for fetal deaths

Population-based congenital abnormality registers form an important data source for the birth prevalence of many congenital disorders (Moorthie et al. 2017). However, as only a minority of single gene disorders are clinically recognisable at birth, and diagnosis in the remainder usually requires specialist diagnostic facilities, only those conditions that cause physical abnormalities are captured in these registers. The published literature was therefore reviewed for alternative prevalence data sources. Three ‘classical’ population-based studies and one review of the collective prevalence of single gene disorders were identified (Ash et al. 1977; Baird et al. 1988; Stevenson 1959; Trimble and Doughty 1974) (see Online resource p2). These studies were set in Northern European or North American populations and reported broadly similar results. They all date prior to 1990, as recent research has tended to focus on basic science only. In the absence of more recent data on birth prevalence, MGDb uses the rates of Baird et al. (1988), based on the British Colombia Health Registry, to represent global baseline birth prevalence of early-onset single gene disorders as these provide the most recent and complete data. However, they apply only for live births and do not include fetal deaths. As no readily accessible data on fetal death associated with early-onset autosomal dominant and X-linked disorders could be found, the livebirth rates reported by Baird et al. are assumed to be equal to the total births affected and no further adjustment is undertaken. A similar approach is taken for the ‘genetic type unknown’ category. However, reliable data exists on prevalence of stillbirths related to consanguinity-associated recessive disorders (Bittles and Black 2010b; Bundey and Alam 1993). These data were used to adjust the rates reported by Baird et al. (Baird et al. 1988). The same stillbirth rate is assumed for non-consanguinity-associated recessive disorders (Table 2).

**Table 2:** Model parameters for the estimation of baseline prevalence included in Modell Global Database

Autosomal Dominant	X-linked disorders	Non-consanguinity-related autosomal recessive	Consanguinity-related	Genetic type unknown
Per 1000 total births	Per 1000 total births	Per 1000 total births	Per 1000 total births	Per 1000 total births
1.4	0.053	1.84^a^	6.5 × *F* × 100	1.16

For consanguinity-related and recessive disorders (Bittles and Black 2010b; Bundey and Alam 1993); for all other disorders (Baird et al. 1988)

^a^Equals 1.66 affected livebirths per 1000 births reported plus 10% stillbirths

##### Investigation of effects of paternal age on gene mutation rate

Mutations arise because of uncorrected errors in DNA replication during cell division. In females, relatively few cell divisions occur in the formation of mature oocytes, but as adult males generate sperm life-long, spermatogonial stem cells may have undergone more than 1000 divisions by 60 years of age. An association between paternal age and prevalence of new mutations is therefore to be expected and has long been recognised (Ramasamy et al. 2015; Tuente 1972), although it has proved hard to quantify. This is because new mutations occur on one chromosome only—i.e. they are heterozygous; therefore, only early-onset severe dominant disorders will show a clinical effect in terms of fetal death, early death or disability in the first generation. The birth prevalence of X-linked and recessive disorders reflects the average mutation rate over previous generations. This would only be affected by medium- to long-term changes in parental age distribution.

Previous studies have demonstrated an exponential effect of paternal age on mutation rate, with a more than four-fold increase at paternal age 45–49 years when compared to a baseline of 30–34 years (Modell and Kuliev 1990) (see Online resource p3). There are large inter-country variations in paternal age (United Nations 2015), and hence, substantial differences would be expected in mutation rates, and the baseline prevalence of single gene disorders. Trends in paternal age distribution for countries with available data have shown a reduction in paternal age from the earliest records in 1940 to the mid-1970s, associated with increased availability of family planning services and the reduction in overall family size in these countries (United Nations 2015). However, since that time, average paternal age has increased and estimated mutation rates based on paternal age distributions have rebounded to similar levels estimated in the1940s and 1950s. This suggests the overall long-term effects of the observed oscillations are quite small. No adjustment for the effects of parental age was therefore included (see Online resource p4 for details).

##### Summary

The reported rates from Western Canada were used to estimate the baseline birth prevalence of rare single gene disorders for all countries with an adjustment to include fetal deaths associated with recessive disorders (Baird et al. 1988) (Table 2).

#### 1b—Estimation of consanguinity-related rare single gene disorders

In human genetics, the definition of a consanguineous union is one in which the partners are related as second cousins or closer. That is, they have one or more common ancestors within the preceding three generations. Consanguineous partnership increases the chance that a couple will both carry the same recessive disease variant and be at risk for having affected children; the effect is particularly marked for rare disorders. Therefore, where consanguineous marriage is common, there is an increased birth prevalence of a wide spectrum of rare recessive conditions (Corry 2014). The genetic implications for offspring are expressed as a coefficient of consanguinity (*F*), which describes the proportion of the children’s gene pairs that are identical because they are inherited from a common ancestor. Table 3 shows the commonest types of parental consanguinity and associated coefficients of consanguinity.

**Table 3:** Degrees of parental consanguinity and corresponding coefficient of consanguinity

Relationship of parents	% of genes identical by descent, above population average	Coefficient of consanguinity (*F*)
Double first cousins (D1C):	12.5	0.125
First cousins (1C)	6.25	0.0625
First cousins once removed (1 1/2 C)	3.13	0.0313
Second cousins (2C)	1.56	0.0156
Non-consanguineous	–	> 0.0

Uncle-niece marriage is common in some communities: *F* is the same as for double first cousins. Co-efficient of consanguinity is also referred to as α in some sources, e.g. Bittles and Black 2015

Data source: (Cavalli-Sforza and Bodmer 2013) (see appendix p5)

##### Global data on consanguinity levels

Estimated values of the mean coefficient of consanguinity (*F*) are available for 288 countries and vary from 0.0001 in many developed countries to 0.0332 in Pakistan (Bittles and Black 2015). In MGDb, these estimated values are used to calculate the total percentage of all parent couples who are consanguineous, assuming that around two thirds of all consanguineous parents are first cousins and one-third are more distant relatives (see Online resource p5). To enable comparisons between populations, MGDb uses a coefficient of consanguinity of 0.01 (equivalent to 1% of genes identical by recent descent) as a unit of parental consanguinity.

##### Observational data of birth prevalence of consanguinity-associated disorders

Studies of birth prevalence of consanguinity-associated disorders undertaken in high-income settings with advanced diagnostic facilities, access to optimal care and long-term follow-up have found between 5.6–7.7 consanguinity-affected births per 1000 total births for each unit of parental consanguinity (0.01*F*) (Bittles and Black 2010a; Bittles and Neel 1994; Bundey and Alam 1993; Sheridan et al. 2013) (see Online resource p5).

##### Summary

For the purposes of MGDb, the mid-point of the available observational studies was used, and the birth prevalence of consanguinity-associated disorders was calculated as:

$$ \mathrm{Births}/1000\ \mathrm{of}\ \mathrm{consanguinity}-\mathrm{associated}\ \mathrm{disorders}=\mathrm{Population}\ F\times 100\times 6.5 $$

### Step 2—Estimation of actual birth prevalence

Baseline birth prevalence estimates provide an assessment of the underlying prevalence in the population in the absence of interventions. However, when estimating the actual birth prevalence, the potential effect of the development and expansion of genetic services should be taken into account. Genetic services can provide risk identification and counselling prior to pregnancy, with a potential impact on couples’ reproductive choices, including prenatal diagnosis for at-risk pregnancies when this is feasible, and the option of termination of pregnancy (TOP) where this is available and culturally acceptable. Risk identification may take place *prospectively* prior to an affected birth or *retrospectively* after the diagnosis of an affected child (Fraser 1972).

#### Estimation of access to genetic services

Genetic services encompass diagnostic, therapeutic and counselling services for management of individuals and families affected by a genetic disorder. Information regarding the proportion of the population with access to specialist diagnostic and therapeutic services is required to calculate actual birth prevalence from the total ‘baseline birth prevalence’. Data on access to these services are not routinely available; we therefore developed a method to estimate access to specialist services (Blencowe et al. 2018). Even in settings with high levels of access to specialist services, including genetic testing, access to TOP for diagnosed affected pregnancies is dependent on the legal status, national policy and local clinical practice of TOP for fetal disorders in the country (Blencowe et al. 2018; UN Population Division 2013). For countries with no observational data, it is assumed that genetic counselling and prenatal diagnosis is incorporated into specialist health services as they develop and that these services will only be available to a proportion of those accessing health care. In addition, it is assumed that only women in countries where TOP for fetal anomaly is legal, or there is documented widespread practice, will be able to access prenatal diagnosis with the option of TOP. The maximum possible percentage of pregnancies terminated is calculated, based on the proportion of women estimated to have access to prenatal diagnosis, the legal status of TOP in the country, and the assumption that all women diagnosed with an affected pregnancy and with access to TOP will terminate the pregnancy. See the third paper in this series for full details (Blencowe et al. 2018).

#### Potential effects of risk identification

*Prospective risk identification* depends on the ability to detect carriers before they have any affected children, but until recently, this has been very limited because the diversity of gene variants underlying most single gene disorders made DNA-based carrier screening unrealistic. Carrier screening is therefore currently limited to common disorders detectable by assay of the protein end-product (e.g. Tay-Sachs disease, haemoglobin disorders). At present, mutation-specific DNA-based screening is available only for cystic fibrosis and some disorders that are particularly common in specific population groups, e.g. French Canadians (Mitchell et al. 1996) and Ashkenazi Jews (Ekstein and Katzenstein 2001). Surveillance of the existing screening programmes shows that prospective carrier screening with the option of prenatal diagnosis can lead to an over 90% fall in affected birth prevalence (Modell et al. 2017).

Extended family studies have been used to assess the risk of dominant and X-linked disorders prior to the birth of an affected child. At present, family studies are rarely offered for recessive disorders because their power of detecting risk is very limited in randomly mating populations (Krawczak et al. 2001). However, their power is much increased when consanguineous marriage is common (Ahmed et al. 2002; Khan et al. 2010). The effect of extended family studies on affected birth prevalence is hard to assess, and we identified no reports seeking to quantify this.

Ongoing developments in genomics such as rapid cost-effective exome scanning can overcome current barriers to prospective carrier screening for rare single gene disorders and may lead to a major reduction in their birth prevalence, particularly in high income countries (Ellard et al. 2015; Lazarin and Haque 2016). The combination of developments in genomics and ongoing retrospective carrier screening efforts in consanguineous populations can generate a greater knowledge and understanding of variants associated with rare genetic diseases. In the future, such efforts may inform variants to investigate through prospective carrier screening. However, translation of such findings into clinical practice will require assessment both of the evidence base surrounding screening for such variants and the ethical, legal and social implications of such a programme.

*Retrospective risk identification* enables parents to avoid a second affected birth by limiting further reproduction or using prenatal diagnosis with the option of termination of pregnancy. However, the maximum associated reduction in affected birth prevalence is relatively modest, ranging from around 13% when total fertility rate is 2 to 45% when it is six (Fraser 1972). In practice, the majority of at-risk couples with fewer than two healthy children undertake further pregnancies in the hope of obtaining unaffected children (Petrou et al. 2000; Safari Moradabadi et al. 2015). Access to preimplantation or early pregnancy diagnosis services, with the option of TOP, can aid parents to complete their desired family size whilst avoiding the birth of a second affected child. However, both physical and cultural barriers exist to such services, and only a minority of couples globally can access these (Izquierdo and Berkshire 2010; Melo and Sequeiros 2012; Zhong et al. 2017). The effect of retrospective detection on overall reduction of affected birth prevalence is hence low, especially where average family sizes are 3 or fewer, as in most settings where genetic services are available. See Online resource p6, (Fraser 1972) and the third paper in this series (Blencowe et al. 2018) for further details.

#### Estimation of the effect of risk identification on birth prevalence

Risk identification was assumed to have minimal impact on birth prevalence for early-onset dominant or X-linked conditions. For recessive disorders, including consanguinity-related, the maximum pre-birth reduction was estimated by firstly allocating each country to one of four groups, based on current policy and practice. These groups are retrospective risk information only, retrospective risk information with access to pre-natal diagnosis and TOP, prospective carrier screening only and prospective carrier screening with access to pre-natal diagnosis and TOP. In case of rare single gene disorders, currently, the majority of risk identification is retrospective in all settings. Next, the maximum potential effect of the current policy was estimated based on current total fertility rate, assuming the average at risk couple aims for two unaffected children (see Online resource p7 and (Blencowe et al. 2018) for details). Finally, the maximum potential reduction in birth prevalence in each country was estimated by applying the maximum potential effect of the country’s policy to the sub-set of the population in each country estimated to have access to specialist services (Blencowe et al. 2018).

#### Estimation of actual live- and stillbirths associated with rare single gene disorders

The estimated actual prevalence of affected births, live- and stillborn, in a given country per 1000 total births was estimated as the baseline birth prevalence minus the maximum number of cases averted by risk identification per 1000 total births.

### Step 3—Estimation of child outcomes

#### Child mortality outcomes

All mortality rates are adjusted for background mortality (Moorthie et al. 2017). The number of under-5 deaths is estimated as:

$$ \mathrm{Total}\ \mathrm{under}5\ \mathrm{deaths}=\left(\mathrm{Affected}\ \mathrm{livebirths}\times \mathrm{under}5\ \mathrm{case}\ \mathrm{fatality}\ \mathrm{rate}\right) $$

$$ \mathrm{Adjusted}\ \mathrm{number}\ \mathrm{of}\ \mathrm{under}5\ \mathrm{deaths}=\mathrm{Total}\ \mathrm{under}5\ \mathrm{deaths}-\left(\left(\frac{\mathrm{Total}\ \mathrm{under}5\ \mathrm{deaths}}{100}\right)\times \mathrm{national}\ \mathrm{U}5\mathrm{MR}\right) $$

Robust follow-up data on consanguinity-associated recessive disorders are available. These show an early mortality of 80–90% in the absence of care (Bittles and Black 2010a), and 28% with optimal care (Bundey and Alam 1993).These data are assumed to be representative for rare recessive disorders in general.

Limited or no data on excess mortality with early-onset dominant, X-linked rare single gene disorders or for the group classified as ‘genetic type unknown’ are available. Available evidence from dominant haemoglobin disorders supports the assumption that early-onset dominant disorders have a higher mortality than recessive disorders. X-linked disorders are typically less severe, and hence are assumed to have a lower mortality. No data on survival are available for the group classified as ‘genetic type unknown’. In order to generate a conservative estimate, this group is assumed to have the same mortality risk as X-linked disorders. A summary of case fatality rates used in MGDb is provided in Table 4. Further details are available online (Modell et al. 2017).

**Table 4:** Estimated early case fatality rates for rare single gene disorders in Modell Global Database, % of affected livebirths

	Care level	Dominant	X-linked	Recessive	Genetic type unknown	Consanguinity-associated
% neonatal deaths^1^	No care	36.0	21.0	19.0	21.0	19.0
	Optimal care	20.4	12.0	11.0	12.0	11.0
% infant deaths	No care	60.0	35.0	39.0	35.0	39.0
	Optimal care	34.0	20.0	14.0	20.0	14.0
% under-5 deaths	No care	100.0	40.0	84.0	40.0	84.0
	Optimal care	50.0	25.0	28.0	25.0	28.0

^1^Reliable collective figures for neonatal death are only available for consanguinity-associated and recessive disorders. Rates for other groups are estimated at 60% of the infant mortality rate

#### Child disability outcomes

Whilst allogeneic haematopoietic stem cell transplantation has been used as a curative treatment for beta-thalassaemia and severe sickle cell disorders and there is some promising research on the effect of gene editing, there is currently no definitive cure for any rare single gene disorders (King and Shenoy 2014; Wang and Gao 2014). Therefore, all survivors affected with rare single gene disorders are assumed to have some degree of disability. This ranges from conditions such as phenylketonuria and thalassaemia where affected individuals with continuing access to appropriate medical intervention can be ‘well on treatment’, to severe physical and mental disability for those unable to access care, or with conditions where no treatment currently exists.

#### Longer-term outcomes

The steps shown above provide details of requirements to estimate child outcomes. However, available survival data enables the construction of life-time survival curves, which can be used in MGDb to calculate mean life expectancy and other longer term outcomes (Modell et al. 2017).

### Regional estimates of baseline birth prevalence of rare single gene disorders

**Fig. 2:**
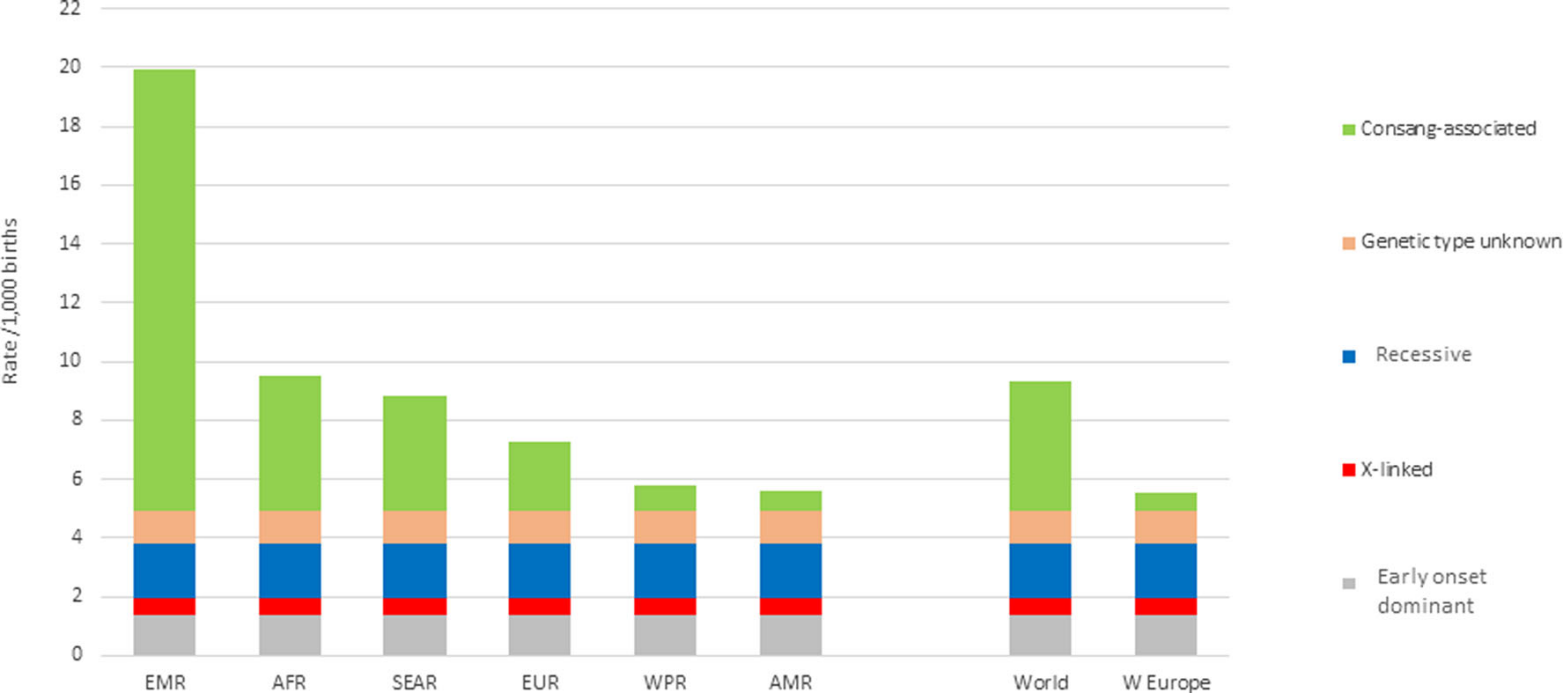
Total Baseline birth prevalence of rare single gene disorders, by WHO region

Figure 2 shows the baseline birth prevalence of rare single gene disorders obtained by applying the above steps to each country and grouping into World Health Organization regions. This figure highlights the important contribution of consanguinity-associated disorders to total rare single-gene disorders.

## Discussion

Any genetic diagnosis (whether in an affected or an unaffected person) involves the family as well as the presenting individual. Relatives need information on the mode of inheritance and possible health and reproductive risks for themselves, access to definitive diagnosis when this is available, and supportive genetic counselling. This requirement, which is specific for single gene disorders, introduces new concepts, and new educational and work force requirements into health services (Samavat and Modell 2004). It is therefore necessary to view these disorders as a coherent group.

Three important points are raised in this article. Firstly, the potential of using existing data to estimate the birth prevalence and outcomes of rare single gene disorders. Secondly, the importance of the contribution of consanguinity-associated disorders to total single gene and overall congenital disorders (Fig. 2). Finally, the potential effect of prospective carrier detection by new methods of genomic analysis, for increasing the currently very limited effect of genetic counselling on the birth prevalence of rare single gene disorders. However, as stated above, this requires consideration of the evidence base in relation to the pathogenicity of particular variants, how they could be incorporated into prospective screening and the ethical, legal and social implications of such a venture.

To date, public health approaches to congenital disorders have tended to focus on congenital anomalies (Black et al. 2010; Liu et al. 2012; Lopez et al. 2006), whilst single gene disorders are seen as too rare, too diverse and too difficult to handle. However, they are an important category of congenital disorder as although individually rare, collectively, they contribute significantly to infant mortality and morbidity (Baird et al. 1954; Emery and Rimoin 1997; Lacaze et al. 2017). One barrier to assessing the disease burden of single gene disorders is that most initiatives for their treatment and/or prevention have been devised by treating clinicians or lay support groups and so tend to be specific to particular disorders. The inevitable focus on individual diagnoses means that the need for patient care obscures their common mode of inheritance and common genetic service needs and tends to favour competition rather than co-operation. The development of the Rare Diseases initiative should help to overcome this difficulty (Dharssi et al. 2017). Though the definition of a rare disease is based on frequency rather than cause, around 80% of recognised rare diseases are in fact single gene disorders. Another barrier is that single gene disorders can affect any aspect of structure or functioning and so are scattered through many categories of the International Classification of Diseases (ICD) (World Health Organization 2010). Therefore, reliance on ICD10 classification can make analysis for this group of disorders very cumbersome. An alternative approach is to apply the basic principles of population genetics to single gene disorders rather than dealing with individual diagnoses, and sufficient information is available to apply this method for assessing their global birth prevalence.

In this paper, we present this non-ICD-based approach to assess the collective epidemiology of rare single gene disorders. A notable limitation is the reliance on few data sources to inform the estimation of their birth prevalence. In recent years there has been a proliferation in epidemiological data regarding single gene disorders in high income settings, with some countries establishing national rare disease registries (www.orpha.net 2017). However, whilst low levels of consistency between studies, poor documentation of methods, confusion between incidence and prevalence and over birth prevalence currently limit the use of these sources for accurate prevalence data. In the future, an adequately funded multi-country umbrella registry organisation could overcome some of these barriers and may provide useful comparable prevalence data for policy-making.

In the absence of reliable data, several assumptions are required, including regarding access to care, and women’s behaviour concerning TOP for an affected pregnancy. These could affect the accuracy of the estimates of actual birth prevalence, e.g. over-estimation of access to pre-natal diagnosis and uptake of TOP would lead to falsely low livebirth prevalence, which may lead to under-provision of care for affected children. Under-estimation of access to optimal care for affected children could result in an over-estimation of single-gene-associated deaths, and an under-estimate of the requirements for ongoing care for those affected living with disability.

In addition, apart from consanguinity-related recessive disorders, data to inform mortality outcomes is limited, often relying on historical data from high-income settings. In the future, data from cohort follow-up studies building on the rare disease registries platforms could provide improved data for high-income settings.

Despite these limitations, this work demonstrates the important role of consanguinity in the prevalence of rare single gene disorders, with around half of all rare single gene disorders globally estimated as being consanguinity-associated (Fig. 2). This shows the need to develop appropriate genetic services to reach those most at risk. Genetic counselling has been shown to have a very limited impact, with around 5% reduction in birth prevalence, when relying on the current retrospective approach (Blencowe et al. 2018; Modell et al. 2017). This compares with an observed 85% reduction in birth prevalence of thalassaemia and 15% reduction for sickle cell disorders in Western Europe, where risk is usually identified prospectively. If prospective risk identification becomes available for the majority of recessive disorders using new techniques such as novel sequencing technologies, and 50% are perceived as severe and 50% as less severe, the experience of haemoglobin disorders suggests that their collective birth prevalence, and associated early mortality and disability, could fall by 50% or more. General deployment of the new diagnostic methods could therefore cause a reduction of around 10% in under-5 deaths, with an even more marked effect on numbers living with disability.

## Conclusion

Rare single gene disorders are an important source of morbidity and premature mortality for affected families. When considered collectively, they account for an important public health burden, which is frequently under-recognised. MGDb provides a method to estimate the burden of these conditions in settings without empirical data, providing population-level estimates that can be used now by programmes and policy makers when planning services. Estimates using this approach will be strengthened in the future as more data become available from a variety of settings to improve the model parameters.

## Electronic supplementary material

ESM 1 (PDF 801 kb)
