# Supplementary material for: Rare single gene disorders: estimating baseline prevalence and outcomes worldwide
Source: J Community Genet. 2018 Aug 14;9(4):397–406. doi: 10.1007/s12687-018-0376-2 (PMC6167259; doi:10.1007/s12687-018-0376-2)
Supplement: Supplementary file 1 — (PDF 801 kb) [file 12687_2018_376_MOESM1_ESM.pdf]

Title: Rare single gene disorders: estimating baseline prevalence and outcomes worldwide

Authors:

Hannah Blencowe, Sowmiya Moorthie, Mary Petrou, Hanan Hamamy, Sue Povey, Alan Bittles, Matthew Darlison, Stephen Gibbons, Bernadette Modell, Congenital Disorders Expert Group

Supplementary on-line appendix

Contents

Observational data of birth prevalence of early-onset single gene disorders 2
The effect of paternal age 3
  Association between paternal age and mutation rate 3
  Paternal-age-related mutation rate in 32 countries in 2012 3
  Paternal-age-related mutation rates in countries with data over time 4
Calculation of percentage of parents that are consanguineous 5
Studies used in the calculation of birth prevalence of consanguinity-associated disorders 5
Calculating the effect of genetic counselling 6
  Prospective risk identification 6
  Retrospective risk identification 6
  Method used in MGDb 6
    Step 1. Allocate each country a policy 6
    Step 2. Calculate the maximum potential effect of genetic counselling in each country 7
    Step 3. Estimate the actual effect of the current policy 7
  References 8

Observational data of birth prevalence of early-onset single gene disorders

Table 1: Classical studies of the collective live birth prevalence of early-onset single gene disorders in Northern European and North American populations

Data Source	Period covered (years)	Dominant, (severe early-onset)	X-linked	Recessive	Genetic type unknown	Total early onset
N. Ireland (Stevenson 1959)	1948-58	2.41	0.4	2.13		4.94
British Columbia (Trimble and Doughty 1974)	1952-72	0.47	0.26	0.73	0.02	1.47
Review of multiple populations (Ash et al. 1977)	Up to 1977	7.00[a]	0.4	2.5		
Birth Colombia (Baird et al. 1988)	1952-83	1.395	0.532	1.655	1.16	4.74

[a] Including late onset

Differences in reported rates are largely due to differences in the range of disorders included and the facilities for accurate diagnosis available at the time.

The “genetic type unknown” category of Baird applies for probable single gene disorders with uncertain mode of inheritance. In the MGDb a conservative approach is taken and these outcomes are assumed to be the same as for X-linked disorders.

The effect of paternal age

Association between paternal age and mutation rate

Classical studies of "sentinel" disorders show an exponential paternal age effect.[1] Figure 1 and Table 2 show a general relationship between paternal age and mutation rate calculated in relative terms (Modell and Kuliev 1990). The curve resembles that for age-related risk of chromosomal non-disjunction in females, but the effect is of a lower order (see Table).

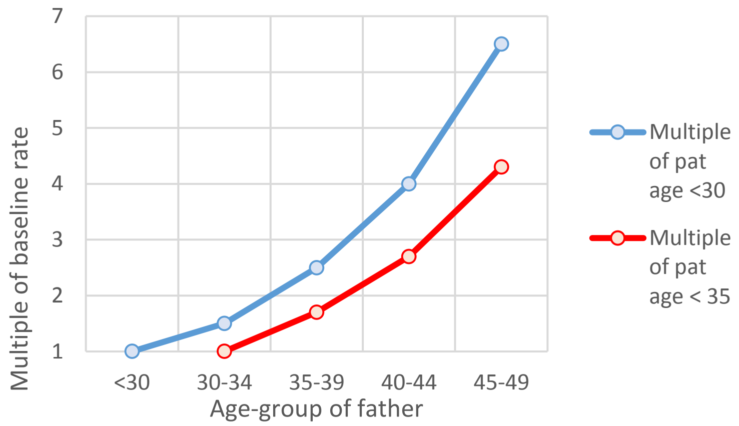

Figure 1. Estimates of relative paternal-age-related mutation rate (Modell and Kuliev 1990).

Table 2. Increase in prevalence of sentinel disorders due to new mutation in relation to paternal age, and comparison with maternal age relationship for Down syndrome.

Paternal age	New mutations		Maternal age	Down syndrome	
	Multiple of paternal age <30	Multiple of paternal age < 35		Multiple of maternal age <30	Multiple of maternal age < 35
<30	1.0		<30	1.0	
30-34	1.5	1.0	30-34	1.8	1.0
35-39	2.5	1.7	35-39	5.7	3.1
40-44	4.0	2.7	40-44	19.6	10.8
45-49	6.5	4.3	45-49	41.9	23.0
50-54			50-54	16.5	9.0

Data source: (Modell and Kuliev 1990).

Paternal-age-related mutation rate in 32 countries in 2012

Figure 2 shows estimated mutation rate as a multiple of rates for fathers less than 30 or 30-34, calculated by applying the rates in Table 2 to UNDY paternal age distribution by 5-year intervals.

[1] The fact that the relationship is exponential rather than linear suggests that an additional age-related factor is involved, e.g. a change in the balance between DNA replication and repair.

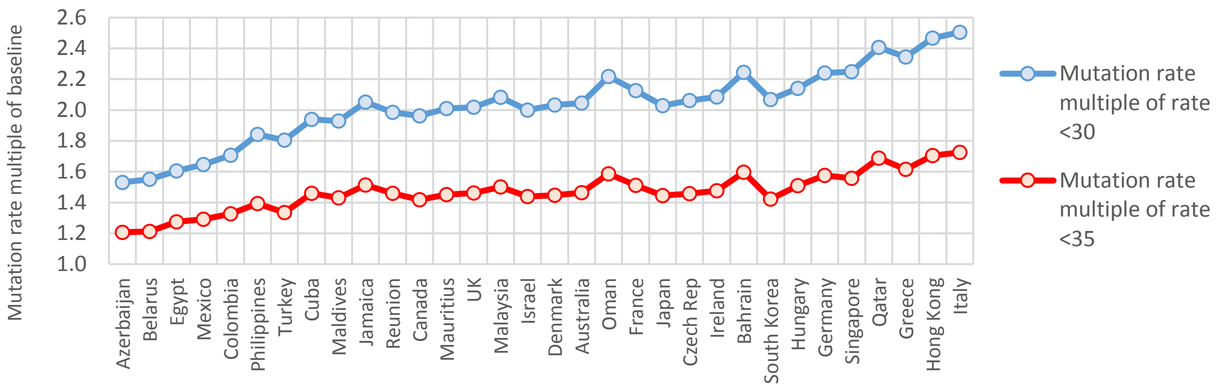

Figure 2. Estimated multiple of baseline paternal-age-related mutation rate in 32 countries in 2012, based on observed % of fathers 30 or 35 plus.

Paternal-age-related mutation rates in countries with data over time

Using the 30 – 34 year group as a baseline, as more than 50% of fathers are aged 30 plus in most countries, in 2012 the paternal-age-related mutation rate may vary by country from 1.2 to 1.7 times this baseline rate. Applying these rates to time-series parental age data for countries with available data shows the earliest estimates were 1.5-1.7 times baseline (Figure 3 and 4). They then fell to around 1.1 times baseline in the 1970s, and have since rebounded to the original level. A similar picture (not shown) was observed for the USA and Chile, and in East Asia.

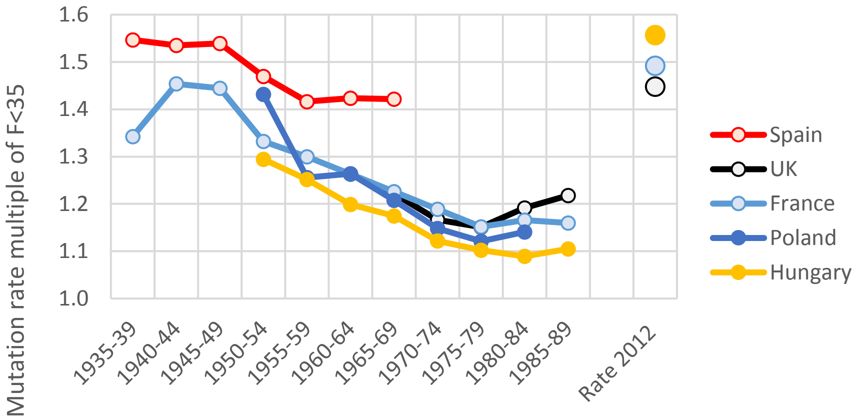

Figure 3. Estimated change in paternal-age-related mutation rate, countries of European region.

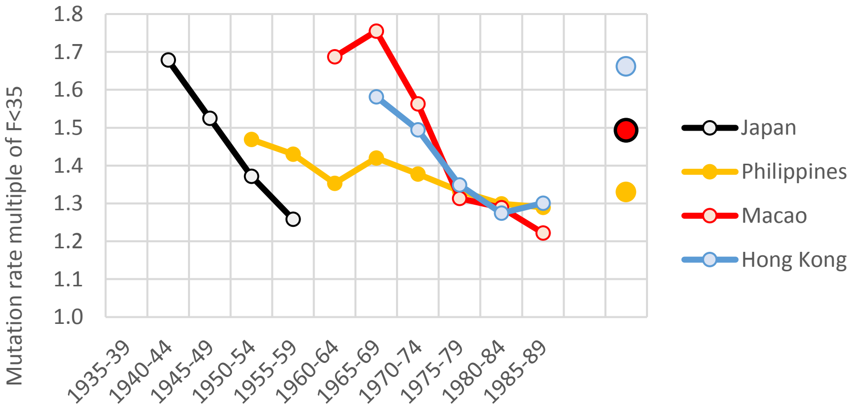

Figure 4. Estimated change in paternal-age-related mutation rate, countries of Western Pacific region.

Calculation of percentage of parents that are consanguineous

A population *F* can be interpreted in terms of equivalent first cousin unions. However, some consanguineous unions are closer than first cousins and many are more distant. The total proportion of unions consanguineous can be roughly estimated by assuming that two-thirds of consanguineous unions are between first cousins and one third between more distant relatives. In MGDb the per cent of unions consanguineous, and the per cent of newborns with consanguineous parents are estimated from population F as follows.

- Population *F* x 100 = population % F
- Population % *F*/0.0625 = equivalent % first cousin unions
- Equivalent % first cousin unions x 1.515 = total % of unions consanguineous, and total % of newborns with consanguineous parents.

The principal sources for data on prevalence of consanguineous marriage are Bittles' online database (Bittles and Black 2015), estimates for Western European countries based on ethnic origin (Modell and Darlison 2008; Modell et al. 2007), and data from Murdock's Ethnographic Atlas (Murdock 1967). Further details can be found in Article 11 of 'A General Method for Establishing the Global Epidemiology of Congenital Disorders' (Modell B et al. 2017).

Studies used in the calculation of birth prevalence of consanguinity-associated disorders

Table 3. Summary of data on birth prevalence of total consanguinity-associated disorders, and birth outcomes in terms of (a) affected births /1,000 and (b) affected /1,000 births per unit of parental consanguinity (0.01*F*).

References	Outcome measured	Comparison	Popul-ation *F*	Consanguinity-associated increment /1,000 births			Consanguinity-associated increment/1,000 per 0.01 F			Stillbirths % of total
				Total	Still-birth	Live birth	Total	Still-birth	Live birth	
(Bittles and Black 2010; Bittles and Neel 1994)	Pre-reproductive mortality (to age 10)	1C versus unrelated	0.0625	44.0	7	37.0	7.0	1.12	5.92	16.0
				35.0	7	28.0	5.6	1.12	4.48	20.0
Birmingham Birth Study (Bundey and Alam 1993)	Congenital disorders to age 5	Pakistani vs white British	0.0431	47.6	5.7	41.9	7.6	0.9	6.70	11.8
Born in Bradford (Sheridan et al. 2013)	Congenital disorders to age 5 (in progress)	Pakistani vs white British	0.0289	22.3	3.5	18.8	7.7	1.2	6.51	15.6
		Pakistani 1C vs unrelated		36.6			5.9			

Calculating the effect of genetic counselling

Prospective risk identification

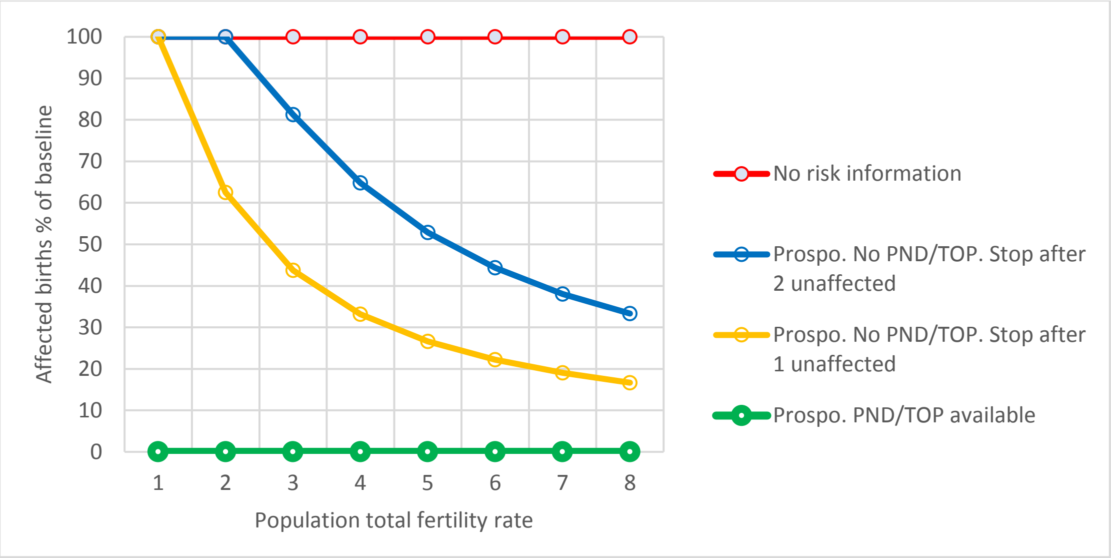

Figure 5 - Maximum possible effect of prospective genetic counselling on affected birth prevalence

Retrospective risk identification

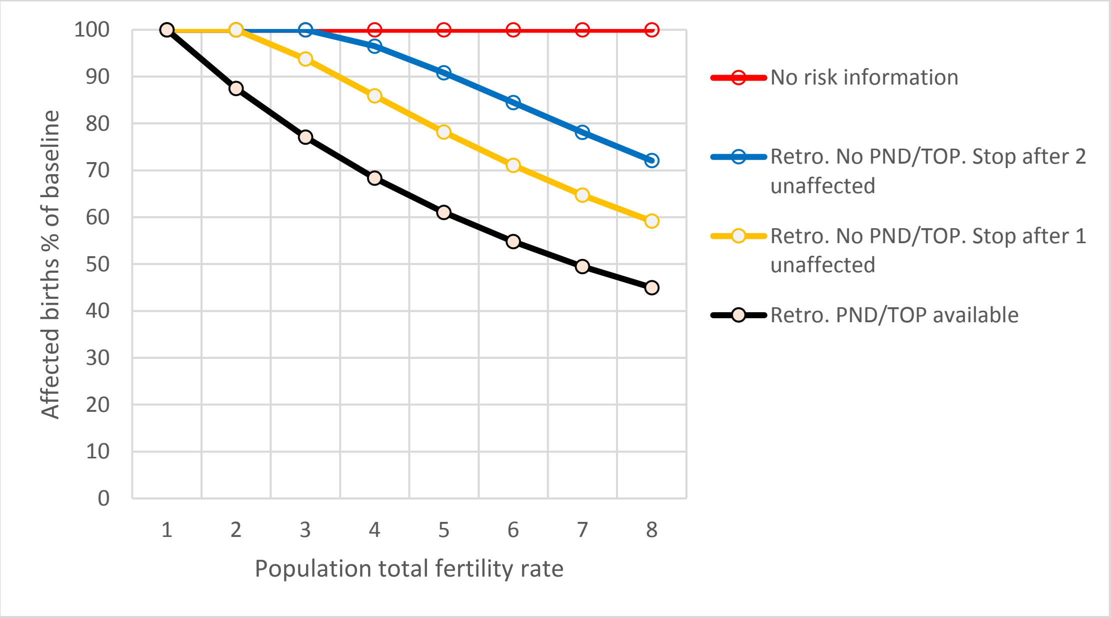

Figure 6 - Maximum possible effect of retrospective genetic counselling on affected birth prevalence

Method used in MGDb

Step 1. Allocate each country a policy

It is assumed that all those with access to specialist care will all receive a diagnosis and will automatically be informed of the recurrence risk as part of the service i.e. retrospective risk information. Each country was allocated to a policy group, based on evidence of the current policy

environment. Evidence of current policy on national carrier screening was based on a web-based review of published information and personal communication with experts working in the country. Evidence of current policy on TOP was based on data on the legal status of TOP collated by the United Nations, unless documented official medical guidance, fatwas or other authoritative documents were available (see (United Nations 2014) and (Blencowe et al. 2018) table 4 and online resource for full details of methods used. Following four policy groupings were used:

1. Retrospective risk information, no prenatal diagnosis (PND)/TOP
2. Retrospective risk information, access to PND/TOP
3. Carrier screening, no access to PND/TOP
4. Carrier screening, access to PND/TOP

Step 2. Calculate the maximum potential effect of genetic counselling in each country

For each country, the maximum potential effect of genetic counselling was estimated based on the current policy and the country's Total Fertility Rate (TFR) (rounded to the nearest whole number), assuming the average at risk couple in each country aims for 2 unaffected (Petrou et al. 2000; Safari Moradabadi et al. 2015)

Table 4 - Maximum possible effect of genetic counselling on affected birth prevalence in relation to population norm for final family size. (% of expectation if no risk information)

Population average final family size	No risk information	Retrospective risk information			Prospective risk information		
		PND/TOP available *	Stop after 1 unaffected	Stop after 2 unaffected	PND/TOP available	Stop after 1 unaffected	Stop after 2 unaffected
1	100	100	100	100	0	100	100
2	100	88	100	100	0	63	100
3	100	77	94	100	0	44	81
4	100	68	86	96	0	33	65
5	100	61	78	91	0	27	53
6	100	55	71	84	0	22	44
7	100	50	65	78	0	19	38
8	100	45	59	72	0	17	33

*The effect on affected birth prevalence if all couples stop after the first affected, or all couples use PND/TOP in pregnancies undertaken after the first affected.

Step 3. Estimate the actual effect of the current policy

The maximum potential reduction in birth prevalence in each country was estimated by applying the maximum potential effect to the sub-set of the population in each country estimated to have access to specialist services (see (Blencowe et al. 2018) for full details of methods used to estimate access to specialist services).

References

Ash P, Vennart J, Carter CO (1977) The incidence of hereditary disease in man Lancet 1:849-851

Baird PA, Anderson TW, Newcombe HB, Lowry RB (1988) Genetic disorders in children and young adults: a population study Am J Hum Genet 42:677-693

Bittles AH, Black ML (2010) Evolution in health and medicine Sackler colloquium: Consanguinity, human evolution, and complex diseases Proc Natl Acad Sci U S A 107:1779-1786. doi: 1710.1073/pnas.0906079106. Epub 0906072009 Sep 0906079123.

Bittles AH, Black ML (2015) Global Patterns and Tables of Consanguinity http://consangnet

Bittles AH, Neel JV (1994) The costs of human inbreeding and their implications for variations at the DNA level Nat Genet 8:117-121. doi: 110.1038/ng1094-1117.

Blencowe H, Moorthie S, Darlinson M, Gibbons S, Modell B (2018) Access to care and the effect of interventions on the outcomes of congenital disorders Journal of Community Genetics (in press)

Bundey S, Alam H (1993) A five-year prospective study of the health of children in different ethnic groups, with particular reference to the effect of inbreeding Eur J Hum Genet 1:206-219.

Modell B, Darlison M, Moorthie S et al (2017) A General Method for Establishing the Global Epidemiology of Congenital Disorders UCL repository http://discoveryuclacuk/1532179/

Modell B, Darlison M (2008) Global epidemiology of haemoglobin disorders and derived service indicators Bull World Health Organ 86:480-487

Modell B et al. (2007) Epidemiology of haemoglobin disorders in Europe: an overview Scand J Clin Lab Invest 67:39-69 doi:10.1080/00365510601046557

Modell B, Kuliev A (1990) Changing paternal age distribution and the human mutation rate in Europe Human genetics 86:198-202

Murdock GP (1967) Ethnographic atlas. Univ. of Pittsburgh, [S.l.]

Petrou M, Modell B, Shetty S, Khan M, Ward RH (2000) Long-term effect of prospective detection of high genetic risk on couples' reproductive life: data for thalassaemia Prenat Diagn 20:469-474

Safari Moradabadi A, Alavi A, Eqbal Eftekhaari T, Dadipoor S (2015) The Reproductive Behavior of Families with Thalassemic Children in Hormozgan Journal of reproduction & infertility 16:167-170

Sheridan E et al. (2013) Risk factors for congenital anomaly in a multiethnic birth cohort: an analysis of the Born in Bradford study Lancet 382:1350-1359 doi:10.1016/S0140-6736(13)61132-0

Stevenson AC (1959) The Load of hereditary defects in human populations Radiation Research Supplement 1:306-325

Trimble BK, Doughty JH (1974) The amount of hereditary disease in human populations Annals of human genetics 38:199-223

United Nations DoEaSA, Population Division, (2014) Abortion Policies and Reproductive Health around the World (United Nations publication, Sales No. E.14.XIII.11).
